# (2*E*)-3-(4-Chloro­phen­yl)-1-(2,4-dimethyl­quinolin-3-yl)prop-2-en-1-one

**DOI:** 10.1107/S1600536811007835

**Published:** 2011-03-05

**Authors:** R. Prasath, P. Bhavana, Seik Weng Ng, Edward R. T. Tiekink

**Affiliations:** a Department of Chemistry, BITS, Pilani - K. K. Birla Goa Campus, Goa 403 726, India; b Department of Chemistry, University of Malaya, 50603 Kuala Lumpur, Malaysia

## Abstract

Two independent mol­ecules comprise the asymmetric unit of the title compound, C_20_H_16_ClNO, which differ in the orientation of the chalcone residue with respect to the quinoline ring [the C—C—C(=O)—C torsion angles are 69.5 (2) and 86.0 (2)°]. The configuration about each of the ethyl­ene bonds [1.342 (2) and 1.338 (2) Å] is *E*. The three-dimensional crystal structure is stabilized by a combination of C—H⋯O, C—H⋯N, C—H⋯π inter­actions and π–π contacts between the independent mol­ecules [*Cg*(C_6_ of quinoline)⋯*Cg*(C_6_ of quinoline) = 3.6719 (11) Å].

## Related literature

For background details and biological applications of quinolines, see: Markees *et al.* (1970 ▶); Campbell *et al.* (1998 ▶); Kalluraya & Sreenivasa (1998 ▶). For the biological activity of chalcones, see: Dimmock *et al.* (1999 ▶); Xiang *et al.* (2006 ▶). For related structures, see: Prasath *et al.* (2010 ▶); Kaiser *et al.* (2009 ▶).
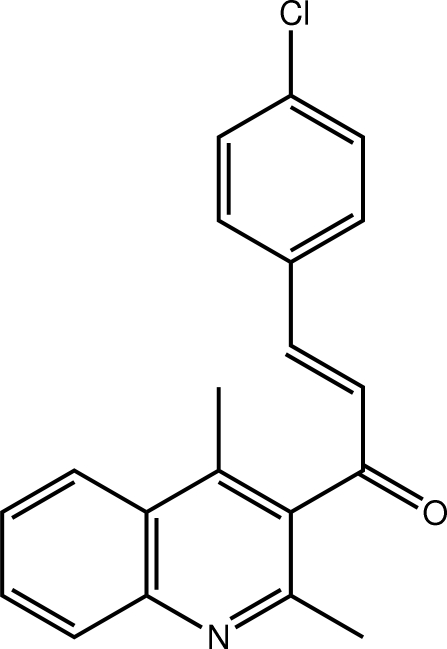


## Experimental

### 

#### Crystal data

C_20_H_16_ClNO
*M* *_r_* = 321.79
Triclinic, 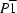

*a* = 11.3172 (5) Å
*b* = 12.0268 (4) Å
*c* = 12.6634 (5) Å
α = 111.318 (3)°
β = 91.620 (3)°
γ = 94.581 (3)°
*V* = 1597.50 (11) Å^3^
*Z* = 4
Mo *K*α radiation
μ = 0.24 mm^−1^
*T* = 100 K
0.30 × 0.20 × 0.10 mm

#### Data collection

Agilent SuperNova Dual diffractometer with an Atlas detector
Absorption correction: multi-scan (*CrysAlis PRO*; Agilent, 2010 ▶) *T*_min_ = 0.931, *T*_max_ = 0.976
13075 measured reflections
6879 independent reflections
5750 reflections with *I* > 2σ(*I*)
*R*_int_ = 0.023

#### Refinement

*R*[*F* ^2^ > 2σ(*F* ^2^)] = 0.038
*wR*(*F* ^2^) = 0.109
*S* = 1.08
6879 reflections
419 parameters
H-atom parameters constrained
Δρ_max_ = 0.30 e Å^−3^
Δρ_min_ = −0.30 e Å^−3^

### 

Data collection: *CrysAlis PRO* (Agilent, 2010 ▶); cell refinement: *CrysAlis PRO*; data reduction: *CrysAlis PRO*; program(s) used to solve structure: *SHELXS97* (Sheldrick, 2008 ▶); program(s) used to refine structure: *SHELXL97* (Sheldrick, 2008 ▶); molecular graphics: *ORTEP-3* (Farrugia, 1997 ▶), *DIAMOND* (Brandenburg, 2006 ▶), Qmol (Gans & Shalloway, 2001 ▶); software used to prepare material for publication: *publCIF* (Westrip, 2010 ▶).

## Supplementary Material

Crystal structure: contains datablocks general, I. DOI: 10.1107/S1600536811007835/hg5005sup1.cif

Structure factors: contains datablocks I. DOI: 10.1107/S1600536811007835/hg5005Isup2.hkl

Additional supplementary materials:  crystallographic information; 3D view; checkCIF report

## Figures and Tables

**Table 1:** Hydrogen-bond geometry (Å, °) *Cg*1 is the centroid of the C35–C40 ring.

*D*—H⋯*A*	*D*—H	H⋯*A*	*D*⋯*A*	*D*—H⋯*A*
C14—H14⋯N2	0.95	2.60	3.500 (2)	158
C20—H20⋯O2^i^	0.95	2.46	3.317 (2)	150
C34—H34⋯N1	0.95	2.51	3.369 (2)	151
C19—H19⋯*Cg*1^ii^	0.95	2.59	3.3826 (17)	142

Symmetry codes: (i) 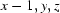; (ii) 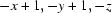.

**Fig. 1.**
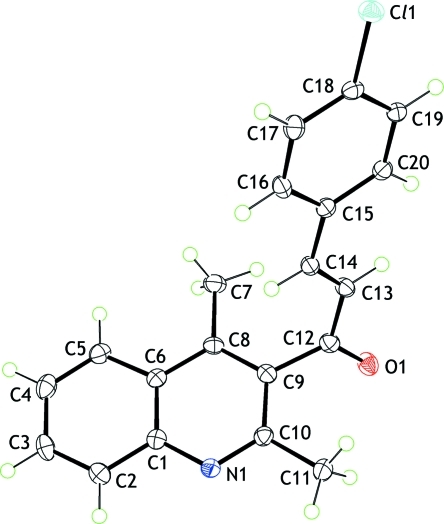
The molecular structure of the first independent molecule in (I) showing the atom-labelling scheme and displacement ellipsoids at the 50% probability level.

**Fig. 2.**
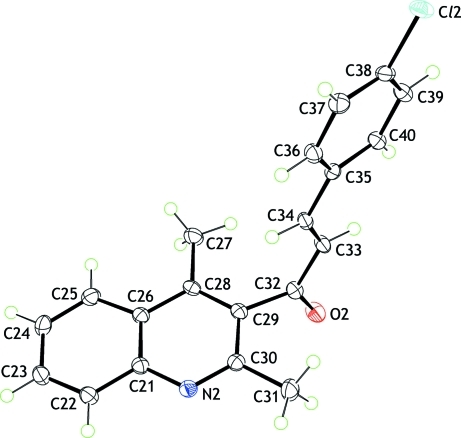
The molecular structure of the second independent molecule in (I) showing the atom-labelling scheme and displacement ellipsoids at the 50% probability level.

**Fig. 3.**
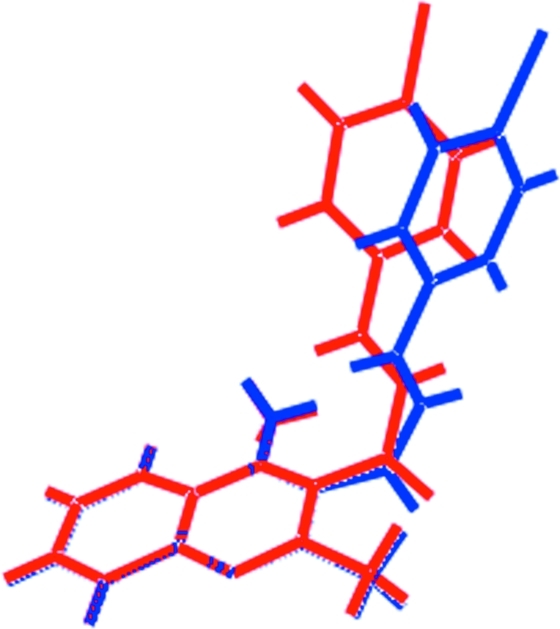
Overlay diagram of the two independent molecules in (I) with the the first independent molecule shown in red.

**Fig. 4.**
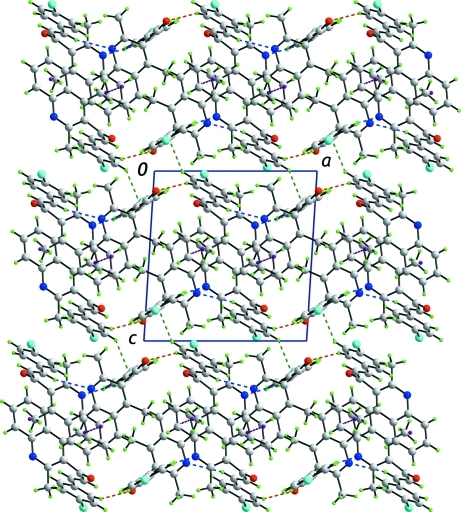
A view in projection down the a axis of the packing in (I). The C—H···N, C—H···O, C—H···π, and π···π interactions are shown as blue, orange, green and purple dashed lines, respectively.

## supplementary crystallographic information

### Comment

Quinoline derivatives find importance owing to their wide occurrence in natural products and in biologically active compounds (Markees *et al.*, 1970; Campbell *et al.*, 1998; Kalluraya & Sreenivasa, 1998). Quinoline chalcone analogues have also attracted significant attention as a result of their bio-activity, *e.g*. anti-plasmodial, anti-microbial, anti-malarial and anti-cancer (Dimmock *et al.*, 1999; Xiang *et al.*, 2006). In continuation of structural studies of these derivatives (Kaiser *et al.*, 2009; Prasath *et al.*, 2010), the title compound, (I), was investigated.

Two independent molecules comprise the asymmetric unit of (I), Figs 1 and 2. Each features an *E* configuration about the C═C bond [C13═C14 is 1.342 (2) Å and C33═C34 is 1.338 (2) Å]. Differences between the independent molecules are highlighted in the overlay diagram, Fig. 3, and relate to the twist about the C9—C12 [C29—C32] bond as seen in the C8—C9—C12—C13 and C28—C29—C32—C33 torsion angles of 69.5 (2) and 86.0 (2) °, respectively.

Molecules are consolidated in the crystal packing by a combination of C—H···O, C—H···N, and C—H···π interactions as detailed in Table 1 as well as π···π contacts, with shortest contact of this type occurring between the two independent molecules [*Cg*(C1–C6)···*Cg*(C21–C26) = 3.6719 (11) Å, angle between rings = 1.27 (9) °], Fig. 4.

### Experimental

A mixture of 3-acetyl-2,4-dimethylquinoline (0.01 *M*), 4-chlorobenzaldehyde (0.01 *M*) and a catalytic amount of KOH in distilled ethanol was stirred for 12 h at room temperature. The resulting mixture was neutralized with dilute acetic acid. The resultant solid was filtered, dried and purified by column chromatography using a 1:3 mixture of ethyl acetate and hexane. Re-crystallization was by slow evaporation of acetone solution of (I) which yielded colourless prisms in 86% yield; *M*.pt. 426–428 K.

### Refinement

The C-bound H atoms were geometrically placed (C–H = 0.95–0.98 Å) and refined as riding with *U_iso_*(H) = 1.2–1.5*U_eq_*(C).

### Crystal data

**Table:**

C_20_H_16_ClNO	*Z* = 4
*M**_r_* = 321.79	*F*(000) = 672
Triclinic, *P*1	*D*_x_ = 1.338 Mg m^−^^3^
Hall symbol: -P 1	Mo *K*α radiation, λ = 0.71073 Å
*a* = 11.3172 (5) Å	Cell parameters from 6658 reflections
*b* = 12.0268 (4) Å	θ = 2.4–29.2°
*c* = 12.6634 (5) Å	µ = 0.24 mm^−^^1^
α = 111.318 (3)°	*T* = 100 K
β = 91.620 (3)°	Prismatic, colourless
γ = 94.581 (3)°	0.30 × 0.20 × 0.10 mm
*V* = 1597.50 (11) Å^3^	

### Data collection

**Table:**

Agilent SuperNova Dual diffractometer with an Atlas detector	6879 independent reflections
Radiation source: SuperNova (Mo) X-ray Source	5750 reflections with *I* > 2σ(*I*)
Mirror	*R*_int_ = 0.023
Detector resolution: 10.4041 pixels mm^-1^	θ_max_ = 27.0°, θ_min_ = 2.4°
ω scan	*h* = −11→14
Absorption correction: multi-scan (*CrysAlis PRO*; Agilent, 2010)	*k* = −14→15
*T*_min_ = 0.931, *T*_max_ = 0.976	*l* = −16→16
13075 measured reflections	

### Refinement

**Table:**

Refinement on *F*^2^	Primary atom site location: structure-invariant direct methods
Least-squares matrix: full	Secondary atom site location: difference Fourier map
*R*[*F*^2^ > 2σ(*F*^2^)] = 0.038	Hydrogen site location: inferred from neighbouring sites
*wR*(*F*^2^) = 0.109	H-atom parameters constrained
*S* = 1.08	*w* = 1/[σ^2^(*F*_o_^2^) + (0.0488*P*)^2^ + 0.5056*P*] where *P* = (*F*_o_^2^ + 2*F*_c_^2^)/3
6879 reflections	(Δ/σ)_max_ = 0.001
419 parameters	Δρ_max_ = 0.30 e Å^−^^3^
0 restraints	Δρ_min_ = −0.30 e Å^−^^3^

### Special details

**Table:**

Geometry. All e.s.d.'s (except the e.s.d. in the dihedral angle between two l.s. planes) are estimated using the full covariance matrix. The cell e.s.d.'s are taken into account individually in the estimation of e.s.d.'s in distances, angles and torsion angles; correlations between e.s.d.'s in cell parameters are only used when they are defined by crystal symmetry. An approximate (isotropic) treatment of cell e.s.d.'s is used for estimating e.s.d.'s involving l.s. planes.
Refinement. Refinement of *F*^2^ against ALL reflections. The weighted *R*-factor *wR* and goodness of fit *S* are based on *F*^2^, conventional *R*-factors *R* are based on *F*, with *F* set to zero for negative *F*^2^. The threshold expression of *F*^2^ > σ(*F*^2^) is used only for calculating *R*-factors(gt) *etc*. and is not relevant to the choice of reflections for refinement. *R*-factors based on *F*^2^ are statistically about twice as large as those based on *F*, and *R*- factors based on ALL data will be even larger.

### Fractional atomic coordinates and isotropic or equivalent isotropic displacement parameters (Å^2^)

**Table:**

	*x*	*y*	*z*	*U*_iso_*/*U*_eq_	
Cl1	0.30818 (4)	1.10668 (4)	0.01940 (3)	0.02200 (11)	
Cl2	0.94188 (4)	−0.07691 (3)	0.17947 (4)	0.02391 (11)	
O1	0.27425 (11)	0.43144 (11)	0.19111 (10)	0.0237 (3)	
O2	1.01594 (11)	0.65990 (10)	0.11770 (10)	0.0244 (3)	
N1	0.63923 (12)	0.47181 (12)	0.31920 (11)	0.0181 (3)	
N2	0.69353 (12)	0.77627 (12)	0.28121 (11)	0.0183 (3)	
C1	0.65234 (14)	0.55428 (14)	0.42863 (13)	0.0169 (3)	
C2	0.75852 (15)	0.56068 (15)	0.49298 (14)	0.0208 (4)	
H2	0.8172	0.5082	0.4604	0.025*	
C3	0.77728 (16)	0.64175 (16)	0.60164 (14)	0.0240 (4)	
H3	0.8489	0.6454	0.6443	0.029*	
C4	0.69125 (17)	0.71968 (16)	0.65055 (14)	0.0255 (4)	
H4	0.7052	0.7762	0.7260	0.031*	
C5	0.58725 (16)	0.71496 (15)	0.59032 (14)	0.0212 (4)	
H5	0.5295	0.7679	0.6247	0.025*	
C6	0.56483 (15)	0.63183 (14)	0.47703 (13)	0.0164 (3)	
C7	0.36198 (16)	0.70116 (16)	0.46065 (15)	0.0230 (4)	
H7A	0.2937	0.6807	0.4054	0.034*	
H7B	0.3912	0.7856	0.4806	0.034*	
H7C	0.3378	0.6877	0.5291	0.034*	
C8	0.45920 (15)	0.62335 (14)	0.40959 (13)	0.0168 (3)	
C9	0.44899 (14)	0.54124 (14)	0.29911 (13)	0.0167 (3)	
C10	0.54205 (15)	0.46604 (14)	0.25775 (13)	0.0178 (3)	
C11	0.53295 (16)	0.37519 (16)	0.13797 (14)	0.0268 (4)	
H11A	0.6084	0.3395	0.1209	0.040*	
H11B	0.5154	0.4148	0.0848	0.040*	
H11C	0.4691	0.3122	0.1303	0.040*	
C12	0.34040 (15)	0.52485 (15)	0.22153 (13)	0.0185 (3)	
C13	0.31533 (15)	0.61965 (15)	0.17951 (13)	0.0193 (3)	
H13	0.2394	0.6139	0.1428	0.023*	
C14	0.39258 (14)	0.71409 (14)	0.18947 (13)	0.0169 (3)	
H14	0.4673	0.7207	0.2284	0.020*	
C15	0.37082 (14)	0.80785 (14)	0.14499 (13)	0.0163 (3)	
C16	0.45856 (15)	0.90306 (15)	0.16371 (13)	0.0185 (3)	
H16	0.5317	0.9044	0.2030	0.022*	
C17	0.44053 (15)	0.99568 (15)	0.12578 (14)	0.0202 (3)	
H17	0.5002	1.0603	0.1395	0.024*	
C18	0.33394 (15)	0.99194 (14)	0.06763 (13)	0.0172 (3)	
C19	0.24596 (15)	0.89808 (14)	0.04630 (13)	0.0175 (3)	
H19	0.1737	0.8965	0.0055	0.021*	
C20	0.26433 (15)	0.80688 (14)	0.08494 (13)	0.0176 (3)	
H20	0.2041	0.7426	0.0707	0.021*	
C21	0.73255 (15)	0.84513 (14)	0.39108 (13)	0.0169 (3)	
C22	0.65761 (15)	0.92923 (15)	0.45675 (14)	0.0214 (4)	
H22	0.5828	0.9355	0.4246	0.026*	
C23	0.69216 (16)	1.00162 (15)	0.56633 (14)	0.0234 (4)	
H23	0.6414	1.0582	0.6096	0.028*	
C24	0.80236 (17)	0.99265 (16)	0.61511 (14)	0.0248 (4)	
H24	0.8256	1.0430	0.6913	0.030*	
C25	0.87611 (16)	0.91200 (15)	0.55367 (14)	0.0223 (4)	
H25	0.9503	0.9067	0.5877	0.027*	
C26	0.84371 (14)	0.83584 (14)	0.43949 (13)	0.0165 (3)	
C27	1.03653 (16)	0.73737 (16)	0.41847 (15)	0.0240 (4)	
H27A	1.0757	0.6761	0.3601	0.036*	
H27B	1.0249	0.7130	0.4837	0.036*	
H27C	1.0862	0.8140	0.4430	0.036*	
C28	0.91775 (14)	0.75128 (14)	0.37029 (13)	0.0166 (3)	
C29	0.87938 (14)	0.68731 (13)	0.25941 (13)	0.0156 (3)	
C30	0.76478 (15)	0.70176 (14)	0.21780 (13)	0.0172 (3)	
C31	0.72207 (17)	0.63121 (17)	0.09640 (14)	0.0266 (4)	
H31A	0.6385	0.6425	0.0851	0.040*	
H31B	0.7301	0.5459	0.0790	0.040*	
H31C	0.7698	0.6592	0.0459	0.040*	
C32	0.96148 (14)	0.61323 (14)	0.17584 (13)	0.0177 (3)	
C33	0.97704 (15)	0.48990 (14)	0.16405 (14)	0.0187 (3)	
H33	1.0385	0.4512	0.1195	0.022*	
C34	0.90871 (14)	0.42888 (14)	0.21311 (13)	0.0164 (3)	
H34	0.8480	0.4693	0.2576	0.020*	
C35	0.91928 (14)	0.30507 (14)	0.20433 (13)	0.0153 (3)	
C36	0.83251 (15)	0.24830 (15)	0.24922 (14)	0.0191 (3)	
H36	0.7681	0.2907	0.2845	0.023*	
C37	0.83849 (15)	0.13140 (15)	0.24336 (14)	0.0209 (4)	
H37	0.7794	0.0940	0.2746	0.025*	
C38	0.93268 (15)	0.07030 (14)	0.19082 (13)	0.0186 (3)	
C39	1.02011 (15)	0.12443 (14)	0.14643 (14)	0.0193 (3)	
H39	1.0844	0.0816	0.1115	0.023*	
C40	1.01375 (14)	0.24142 (14)	0.15301 (13)	0.0175 (3)	
H40	1.0738	0.2786	0.1225	0.021*	

### Atomic displacement parameters (Å^2^)

**Table:**

	*U*^11^	*U*^22^	*U*^33^	*U*^12^	*U*^13^	*U*^23^
Cl1	0.0232 (2)	0.0193 (2)	0.0263 (2)	0.00249 (16)	0.00071 (17)	0.01157 (17)
Cl2	0.0277 (2)	0.0146 (2)	0.0308 (2)	0.00188 (17)	−0.00441 (18)	0.01043 (17)
O1	0.0212 (6)	0.0235 (6)	0.0280 (6)	−0.0027 (5)	−0.0042 (5)	0.0127 (5)
O2	0.0264 (7)	0.0203 (6)	0.0300 (6)	0.0040 (5)	0.0104 (5)	0.0123 (5)
N1	0.0164 (7)	0.0177 (7)	0.0191 (7)	0.0026 (6)	0.0009 (6)	0.0051 (6)
N2	0.0168 (7)	0.0192 (7)	0.0183 (7)	0.0023 (6)	−0.0008 (6)	0.0063 (6)
C1	0.0179 (8)	0.0165 (8)	0.0176 (8)	−0.0006 (6)	0.0019 (6)	0.0081 (6)
C2	0.0177 (8)	0.0245 (9)	0.0228 (8)	0.0016 (7)	0.0020 (7)	0.0118 (7)
C3	0.0221 (9)	0.0320 (10)	0.0193 (8)	−0.0018 (8)	−0.0047 (7)	0.0126 (7)
C4	0.0339 (10)	0.0244 (9)	0.0163 (8)	−0.0014 (8)	−0.0017 (7)	0.0062 (7)
C5	0.0273 (9)	0.0193 (8)	0.0179 (8)	0.0040 (7)	0.0030 (7)	0.0072 (7)
C6	0.0194 (8)	0.0143 (7)	0.0172 (7)	0.0000 (6)	0.0026 (6)	0.0080 (6)
C7	0.0242 (9)	0.0235 (9)	0.0226 (8)	0.0084 (7)	0.0033 (7)	0.0087 (7)
C8	0.0194 (8)	0.0154 (8)	0.0187 (8)	0.0029 (6)	0.0026 (7)	0.0096 (6)
C9	0.0159 (8)	0.0167 (8)	0.0196 (8)	0.0001 (6)	0.0007 (6)	0.0095 (7)
C10	0.0178 (8)	0.0159 (8)	0.0188 (8)	0.0003 (6)	0.0010 (7)	0.0055 (6)
C11	0.0228 (9)	0.0270 (9)	0.0222 (9)	0.0059 (8)	−0.0029 (7)	−0.0012 (7)
C12	0.0165 (8)	0.0209 (8)	0.0185 (8)	0.0035 (7)	0.0032 (7)	0.0073 (7)
C13	0.0162 (8)	0.0222 (8)	0.0194 (8)	0.0050 (7)	−0.0001 (7)	0.0071 (7)
C14	0.0157 (8)	0.0202 (8)	0.0135 (7)	0.0050 (7)	0.0011 (6)	0.0041 (6)
C15	0.0167 (8)	0.0180 (8)	0.0133 (7)	0.0040 (6)	0.0030 (6)	0.0041 (6)
C16	0.0152 (8)	0.0234 (8)	0.0165 (8)	0.0026 (7)	−0.0006 (6)	0.0067 (7)
C17	0.0183 (8)	0.0213 (8)	0.0199 (8)	−0.0014 (7)	0.0004 (7)	0.0071 (7)
C18	0.0205 (8)	0.0150 (8)	0.0164 (7)	0.0044 (6)	0.0038 (6)	0.0053 (6)
C19	0.0168 (8)	0.0166 (8)	0.0171 (8)	0.0042 (7)	0.0005 (6)	0.0032 (6)
C20	0.0181 (8)	0.0160 (8)	0.0173 (8)	0.0016 (6)	0.0013 (7)	0.0043 (6)
C21	0.0186 (8)	0.0149 (8)	0.0183 (8)	0.0017 (6)	0.0023 (7)	0.0072 (6)
C22	0.0204 (9)	0.0222 (9)	0.0231 (8)	0.0065 (7)	0.0035 (7)	0.0090 (7)
C23	0.0281 (10)	0.0211 (9)	0.0225 (8)	0.0088 (7)	0.0070 (7)	0.0079 (7)
C24	0.0327 (10)	0.0223 (9)	0.0157 (8)	0.0037 (8)	0.0004 (7)	0.0026 (7)
C25	0.0252 (9)	0.0216 (9)	0.0198 (8)	0.0030 (7)	−0.0031 (7)	0.0072 (7)
C26	0.0181 (8)	0.0140 (8)	0.0179 (8)	0.0013 (6)	0.0010 (6)	0.0064 (6)
C27	0.0208 (9)	0.0242 (9)	0.0250 (9)	0.0069 (7)	−0.0032 (7)	0.0058 (7)
C28	0.0166 (8)	0.0130 (7)	0.0217 (8)	0.0022 (6)	0.0017 (7)	0.0082 (6)
C29	0.0171 (8)	0.0117 (7)	0.0190 (8)	0.0005 (6)	0.0024 (6)	0.0068 (6)
C30	0.0183 (8)	0.0161 (8)	0.0178 (8)	0.0010 (7)	0.0010 (7)	0.0070 (6)
C31	0.0247 (9)	0.0284 (10)	0.0199 (8)	0.0049 (8)	−0.0021 (7)	0.0007 (7)
C32	0.0166 (8)	0.0161 (8)	0.0191 (8)	0.0007 (6)	0.0012 (7)	0.0054 (6)
C33	0.0202 (8)	0.0151 (8)	0.0207 (8)	0.0057 (7)	0.0053 (7)	0.0053 (6)
C34	0.0163 (8)	0.0147 (8)	0.0162 (7)	0.0036 (6)	0.0015 (6)	0.0028 (6)
C35	0.0166 (8)	0.0148 (8)	0.0135 (7)	0.0014 (6)	−0.0017 (6)	0.0041 (6)
C36	0.0197 (8)	0.0185 (8)	0.0195 (8)	0.0040 (7)	0.0043 (7)	0.0068 (7)
C37	0.0223 (9)	0.0200 (8)	0.0219 (8)	0.0002 (7)	0.0026 (7)	0.0099 (7)
C38	0.0232 (9)	0.0134 (8)	0.0190 (8)	0.0020 (7)	−0.0054 (7)	0.0060 (6)
C39	0.0164 (8)	0.0156 (8)	0.0239 (8)	0.0036 (6)	−0.0005 (7)	0.0048 (7)
C40	0.0162 (8)	0.0156 (8)	0.0199 (8)	0.0009 (6)	0.0005 (7)	0.0059 (6)

### Geometric parameters (Å, °)

**Table:**

Cl1—C18	1.7404 (16)	C18—C19	1.386 (2)
Cl2—C38	1.7354 (16)	C19—C20	1.381 (2)
O1—C12	1.227 (2)	C19—H19	0.9500
O2—C32	1.2279 (19)	C20—H20	0.9500
N1—C10	1.312 (2)	C21—C22	1.414 (2)
N1—C1	1.375 (2)	C21—C26	1.414 (2)
N2—C30	1.314 (2)	C22—C23	1.369 (2)
N2—C21	1.373 (2)	C22—H22	0.9500
C1—C6	1.412 (2)	C23—C24	1.405 (3)
C1—C2	1.414 (2)	C23—H23	0.9500
C2—C3	1.365 (2)	C24—C25	1.364 (2)
C2—H2	0.9500	C24—H24	0.9500
C3—C4	1.403 (3)	C25—C26	1.420 (2)
C3—H3	0.9500	C25—H25	0.9500
C4—C5	1.370 (3)	C26—C28	1.426 (2)
C4—H4	0.9500	C27—C28	1.507 (2)
C5—C6	1.421 (2)	C27—H27A	0.9800
C5—H5	0.9500	C27—H27B	0.9800
C6—C8	1.424 (2)	C27—H27C	0.9800
C7—C8	1.506 (2)	C28—C29	1.371 (2)
C7—H7A	0.9800	C29—C30	1.433 (2)
C7—H7B	0.9800	C29—C32	1.510 (2)
C7—H7C	0.9800	C30—C31	1.504 (2)
C8—C9	1.383 (2)	C31—H31A	0.9800
C9—C10	1.428 (2)	C31—H31B	0.9800
C9—C12	1.506 (2)	C31—H31C	0.9800
C10—C11	1.508 (2)	C32—C33	1.461 (2)
C11—H11A	0.9800	C33—C34	1.338 (2)
C11—H11B	0.9800	C33—H33	0.9500
C11—H11C	0.9800	C34—C35	1.467 (2)
C12—C13	1.466 (2)	C34—H34	0.9500
C13—C14	1.342 (2)	C35—C40	1.400 (2)
C13—H13	0.9500	C35—C36	1.400 (2)
C14—C15	1.466 (2)	C36—C37	1.388 (2)
C14—H14	0.9500	C36—H36	0.9500
C15—C16	1.400 (2)	C37—C38	1.389 (2)
C15—C20	1.404 (2)	C37—H37	0.9500
C16—C17	1.390 (2)	C38—C39	1.383 (2)
C16—H16	0.9500	C39—C40	1.387 (2)
C17—C18	1.384 (2)	C39—H39	0.9500
C17—H17	0.9500	C40—H40	0.9500
			
C10—N1—C1	118.49 (14)	C15—C20—H20	119.6
C30—N2—C21	118.16 (14)	N2—C21—C22	117.67 (15)
N1—C1—C6	122.40 (14)	N2—C21—C26	122.79 (14)
N1—C1—C2	117.73 (14)	C22—C21—C26	119.54 (14)
C6—C1—C2	119.87 (14)	C23—C22—C21	120.46 (16)
C3—C2—C1	120.43 (16)	C23—C22—H22	119.8
C3—C2—H2	119.8	C21—C22—H22	119.8
C1—C2—H2	119.8	C22—C23—C24	120.31 (15)
C2—C3—C4	120.26 (16)	C22—C23—H23	119.8
C2—C3—H3	119.9	C24—C23—H23	119.8
C4—C3—H3	119.9	C25—C24—C23	120.36 (16)
C5—C4—C3	120.53 (16)	C25—C24—H24	119.8
C5—C4—H4	119.7	C23—C24—H24	119.8
C3—C4—H4	119.7	C24—C25—C26	120.98 (16)
C4—C5—C6	120.72 (16)	C24—C25—H25	119.5
C4—C5—H5	119.6	C26—C25—H25	119.5
C6—C5—H5	119.6	C21—C26—C25	118.34 (14)
C1—C6—C5	118.19 (15)	C21—C26—C28	118.04 (14)
C1—C6—C8	118.38 (14)	C25—C26—C28	123.62 (15)
C5—C6—C8	123.43 (15)	C28—C27—H27A	109.5
C8—C7—H7A	109.5	C28—C27—H27B	109.5
C8—C7—H7B	109.5	H27A—C27—H27B	109.5
H7A—C7—H7B	109.5	C28—C27—H27C	109.5
C8—C7—H7C	109.5	H27A—C27—H27C	109.5
H7A—C7—H7C	109.5	H27B—C27—H27C	109.5
H7B—C7—H7C	109.5	C29—C28—C26	118.19 (14)
C9—C8—C6	118.16 (14)	C29—C28—C27	121.81 (14)
C9—C8—C7	122.28 (15)	C26—C28—C27	119.96 (14)
C6—C8—C7	119.55 (14)	C28—C29—C30	119.89 (14)
C8—C9—C10	119.42 (15)	C28—C29—C32	121.05 (14)
C8—C9—C12	122.13 (14)	C30—C29—C32	118.62 (14)
C10—C9—C12	118.41 (14)	N2—C30—C29	122.82 (14)
N1—C10—C9	123.13 (14)	N2—C30—C31	117.12 (14)
N1—C10—C11	116.29 (14)	C29—C30—C31	120.05 (14)
C9—C10—C11	120.58 (14)	C30—C31—H31A	109.5
C10—C11—H11A	109.5	C30—C31—H31B	109.5
C10—C11—H11B	109.5	H31A—C31—H31B	109.5
H11A—C11—H11B	109.5	C30—C31—H31C	109.5
C10—C11—H11C	109.5	H31A—C31—H31C	109.5
H11A—C11—H11C	109.5	H31B—C31—H31C	109.5
H11B—C11—H11C	109.5	O2—C32—C33	120.45 (14)
O1—C12—C13	119.77 (15)	O2—C32—C29	117.96 (14)
O1—C12—C9	120.40 (15)	C33—C32—C29	121.60 (14)
C13—C12—C9	119.81 (14)	C34—C33—C32	123.07 (15)
C14—C13—C12	124.27 (15)	C34—C33—H33	118.5
C14—C13—H13	117.9	C32—C33—H33	118.5
C12—C13—H13	117.9	C33—C34—C35	125.80 (15)
C13—C14—C15	125.19 (15)	C33—C34—H34	117.1
C13—C14—H14	117.4	C35—C34—H34	117.1
C15—C14—H14	117.4	C40—C35—C36	118.54 (14)
C16—C15—C20	118.31 (15)	C40—C35—C34	122.29 (14)
C16—C15—C14	119.19 (14)	C36—C35—C34	119.16 (14)
C20—C15—C14	122.50 (15)	C37—C36—C35	121.45 (15)
C17—C16—C15	121.19 (15)	C37—C36—H36	119.3
C17—C16—H16	119.4	C35—C36—H36	119.3
C15—C16—H16	119.4	C36—C37—C38	118.57 (15)
C18—C17—C16	118.76 (16)	C36—C37—H37	120.7
C18—C17—H17	120.6	C38—C37—H37	120.7
C16—C17—H17	120.6	C39—C38—C37	121.22 (15)
C17—C18—C19	121.47 (15)	C39—C38—Cl2	118.80 (12)
C17—C18—Cl1	119.97 (13)	C37—C38—Cl2	119.98 (13)
C19—C18—Cl1	118.56 (13)	C38—C39—C40	119.85 (15)
C20—C19—C18	119.38 (15)	C38—C39—H39	120.1
C20—C19—H19	120.3	C40—C39—H39	120.1
C18—C19—H19	120.3	C39—C40—C35	120.35 (15)
C19—C20—C15	120.89 (15)	C39—C40—H40	119.8
C19—C20—H20	119.6	C35—C40—H40	119.8
			
C10—N1—C1—C6	0.5 (2)	C30—N2—C21—C22	176.92 (14)
C10—N1—C1—C2	−178.93 (15)	C30—N2—C21—C26	−2.4 (2)
N1—C1—C2—C3	179.21 (15)	N2—C21—C22—C23	−179.12 (15)
C6—C1—C2—C3	−0.2 (2)	C26—C21—C22—C23	0.2 (2)
C1—C2—C3—C4	0.0 (3)	C21—C22—C23—C24	−0.4 (3)
C2—C3—C4—C5	0.4 (3)	C22—C23—C24—C25	0.2 (3)
C3—C4—C5—C6	−0.5 (3)	C23—C24—C25—C26	0.2 (3)
N1—C1—C6—C5	−179.25 (14)	N2—C21—C26—C25	179.46 (15)
C2—C1—C6—C5	0.2 (2)	C22—C21—C26—C25	0.2 (2)
N1—C1—C6—C8	0.3 (2)	N2—C21—C26—C28	0.2 (2)
C2—C1—C6—C8	179.69 (15)	C22—C21—C26—C28	−179.11 (14)
C4—C5—C6—C1	0.2 (2)	C24—C25—C26—C21	−0.4 (2)
C4—C5—C6—C8	−179.31 (16)	C24—C25—C26—C28	178.87 (16)
C1—C6—C8—C9	−1.4 (2)	C21—C26—C28—C29	2.7 (2)
C5—C6—C8—C9	178.07 (15)	C25—C26—C28—C29	−176.56 (15)
C1—C6—C8—C7	177.33 (14)	C21—C26—C28—C27	−179.45 (15)
C5—C6—C8—C7	−3.2 (2)	C25—C26—C28—C27	1.3 (2)
C6—C8—C9—C10	1.8 (2)	C26—C28—C29—C30	−3.3 (2)
C7—C8—C9—C10	−176.91 (15)	C27—C28—C29—C30	178.82 (15)
C6—C8—C9—C12	179.45 (14)	C26—C28—C29—C32	168.91 (14)
C7—C8—C9—C12	0.7 (2)	C27—C28—C29—C32	−8.9 (2)
C1—N1—C10—C9	−0.1 (2)	C21—N2—C30—C29	1.7 (2)
C1—N1—C10—C11	179.24 (14)	C21—N2—C30—C31	−177.48 (14)
C8—C9—C10—N1	−1.1 (2)	C28—C29—C30—N2	1.2 (2)
C12—C9—C10—N1	−178.82 (15)	C32—C29—C30—N2	−171.27 (14)
C8—C9—C10—C11	179.60 (15)	C28—C29—C30—C31	−179.64 (15)
C12—C9—C10—C11	1.9 (2)	C32—C29—C30—C31	7.9 (2)
C8—C9—C12—O1	−112.18 (18)	C28—C29—C32—O2	−93.64 (19)
C10—C9—C12—O1	65.5 (2)	C30—C29—C32—O2	78.7 (2)
C8—C9—C12—C13	69.5 (2)	C28—C29—C32—C33	86.0 (2)
C10—C9—C12—C13	−112.79 (17)	C30—C29—C32—C33	−101.63 (18)
O1—C12—C13—C14	−166.68 (16)	O2—C32—C33—C34	−170.54 (16)
C9—C12—C13—C14	11.6 (2)	C29—C32—C33—C34	9.8 (3)
C12—C13—C14—C15	177.97 (14)	C32—C33—C34—C35	179.74 (15)
C13—C14—C15—C16	178.60 (15)	C33—C34—C35—C40	7.9 (3)
C13—C14—C15—C20	−0.7 (2)	C33—C34—C35—C36	−172.45 (16)
C20—C15—C16—C17	1.1 (2)	C40—C35—C36—C37	−0.2 (2)
C14—C15—C16—C17	−178.23 (14)	C34—C35—C36—C37	−179.86 (15)
C15—C16—C17—C18	−0.6 (2)	C35—C36—C37—C38	−0.5 (2)
C16—C17—C18—C19	−0.3 (2)	C36—C37—C38—C39	0.9 (2)
C16—C17—C18—Cl1	179.76 (12)	C36—C37—C38—Cl2	−178.83 (13)
C17—C18—C19—C20	0.7 (2)	C37—C38—C39—C40	−0.7 (2)
Cl1—C18—C19—C20	−179.33 (12)	Cl2—C38—C39—C40	179.07 (12)
C18—C19—C20—C15	−0.2 (2)	C38—C39—C40—C35	0.0 (2)
C16—C15—C20—C19	−0.7 (2)	C36—C35—C40—C39	0.5 (2)
C14—C15—C20—C19	178.65 (14)	C34—C35—C40—C39	−179.90 (15)

### Hydrogen-bond geometry (Å, °)

**Table:**

Cg1 is the centroid of the C35–C40 ring.

**Table:**

*D*—H···*A*	*D*—H	H···*A*	*D*···*A*	*D*—H···*A*
C14—H14···N2	0.95	2.60	3.500 (2)	158
C20—H20···O2^i^	0.95	2.46	3.317 (2)	150
C34—H34···N1	0.95	2.51	3.369 (2)	151
C19—H19···Cg1^ii^	0.95	2.59	3.3826 (17)	142

Symmetry codes: (i) *x*−1, *y*, *z*; (ii) −*x*+1, −*y*+1, −*z*.
